# Inhibition of androgen receptor promotes CXC-chemokine receptor 7-mediated prostate cancer cell survival

**DOI:** 10.1038/s41598-017-02918-3

**Published:** 2017-06-08

**Authors:** James J. Hoy, Georgios Kallifatidis, Diandra K. Smith, Bal L. Lokeshwar

**Affiliations:** 1 Sheila and David Fuente Graduate Program in Cancer Biology, University of Miami-Miller School of Medicine, Miami, FL USA; 2 Georgia Cancer Center, Augusta University, Augusta, GA USA; 3 Research Service, Charlie Norwood VA Medical Center, Augusta, GA USA

## Abstract

The atypical C-X-C chemokine receptor 7 (CXCR7) has been implicated in supporting aggressive cancer phenotypes in several cancers including prostate cancer. However, the mechanisms driving overexpression of this receptor in cancer are poorly understood. This study investigates the role of androgen receptor (AR) in regulating CXCR7. Androgen deprivation or AR inhibition significantly increased CXCR7 expression in androgen-responsive prostate cancer cell lines, which was accompanied by enhanced epidermal growth factor receptor (EGFR)-mediated mitogenic signaling, promoting tumor cell survival through an androgen-independent signaling program. Using multiple approaches we demonstrate that AR directly binds to the CXCR7 promoter, suppressing transcription. Clustered regularly interspaced short palindromic repeats (CRISPR) directed Cas9 nuclease-mediated gene editing of CXCR7 revealed that prostate cancer cells depend on CXCR7 for proliferation, survival and clonogenic potential. Loss of CXCR7 expression by CRISPR-Cas9 gene editing resulted in a halt of cell proliferation, severely impaired EGFR signaling and the onset of cellular senescence. Characterization of a mutated CXCR7-expressing LNCaP cell clone showed altered intracellular signaling and reduced spheroid formation potential. Our results demonstrate that CXCR7 is a potential target for adjuvant therapy in combination with androgen deprivation therapy (ADT) to prevent androgen-independent tumor cell survival.

## Introduction

Prostate cancer is among the most common malignancies diagnosed in men worldwide^1^. The five-year survival rate is near 100% with early detection and treatment with either surgery or radiation for localized disease^2–4^. However, approximately 20%–30% of patients develop metastases and therapeutic resistance, leading to lethal castration-resistant prostate cancer (CRPC)^5^. To date, the mechanisms facilitating resistance to androgen-deprivation and anti-AR therapies in prostate cancer remain poorly understood.

Chemokines and their receptors are targets for investigation, due to their involvement in both normal and abnormal physiological behaviors, such as inflammation, immunity, chemotaxis, and metastasis of tumor cells^6–8^. The cysteine-X-cysteine (CXC) motif chemokine recognizing receptors (CXCRs) are a family of 7-transmembrane spanning G-protein coupled receptors (GPCRs) which are involved in driving prostate cancer growth, migration, and survival phenotypes^7, 9^. The most recently discovered member of this family, CXCR7, is an atypical receptor lacking canonical G-protein signaling activation upon ligand binding^10^, but its expression is linked to aggressive tumor phenotypes in several cancer models, including colon cancer^11^ breast cancer^12, 13^, hepatocellular carcinoma^14^ and prostate cancer^7, 15, 16^. CXCR7 has also been identified as a prognostic marker for poor patient outcome in colorectal^17^ and non-small cell lung cancers^18^. Human tissue microarray immunohistochemical staining has revealed significantly increased CXCR7 expression in high grade prostate tumor tissues as well as in metastatic lesions compared to benign hyperplasia^15^. While increased expression of CXCR7 is correlated with aggressive cancer, the mechanisms of CXCR7 dysregulation in prostate cancer and its involvement in therapeutic resistance remain unclear.

During androgen deprivation therapy (ADT), alternative signaling pathways including those mediated by receptor tyrosine kinases (e.g. epidermal growth factor receptor [EGFR]) are activated, supporting androgen-independent survival and proliferation involved in therapeutic resistance^19–21^. We have previously reported that CXCR7 (independent of binding its ligand, stromal cell-derived factor 1α [SDF-1α]) interacts with the epidermal growth factor receptor (EGFR), leading to increased EGF-stimulated EGFR phosphorylation (particularly at tyrosine 1110 [Y1110]), enhanced downstream mitogenic signaling as well as tumor cell proliferation and survival^13, 16^. Based on these findings, we were interested in determining whether CXCR7 is also involved in the signaling cascades that facilitate the transition to CRPC in the context of ADT. The importance of CXCR7 in facilitating androgen deprivation resistance in prostate cancer may be revealed by clarifying this regulatory axis.

This current study investigates the regulatory role of androgen receptor (AR) on CXCR7 transcription in prostate cancer cells. Furthermore, we utilized the recently established clustered regularly interspaced short palindromic repeats (CRISPR)-Cas9 nuclease targeted genomic DNA editing method^22^ to selectively eliminate CXCR7 and investigate the requirement for CXCR7 in potentiating the EGFR signaling axis during ADT.

## Methods

### Cell culture

Human prostate epithelial tumor cell lines LNCaP (American Type Culture Collection [ATCC]; Manassas, VA; CRL-1740) and CRW-22Rv1 (ATCC; CRL-2505) were cultured in RPMI-1640 (Corning cellgro; Corning, NY; 10-040-CV), and C4-2B cells (ViroMed Laboratories; Burlington, NC; 12–103) were cultured in T-medium prepared as described previously^23^; media were supplemented with 10% (5% for T-medium) fetal bovine serum (FBS) (Atlanta Biologicals; Flowery Branch, GA) and 10 μg/mL gentamicin (Sigma-Aldrich; St. Louis, MO). All cell lines were maintained in a humidified incubator at 37 °C and 5% CO_2_ for no more than 10 passages. Cells were regularly tested for mycoplasma contamination with the MycoSensor PCR Assay Kit (Agilent Technologies; Santa Clara, CA; 302108).

For androgen deprivation, cells were incubated in charcoal-dextran treated FBS (CDFBS) supplemented medium for 48 hours for RNA or 72 hours for protein analysis. Androgen stimulation was carried out by pre-incubating cells 48 hours in CDFBS medium, then stimulation with the non-hydrolysable androgen analog, methyltrienolone (R1881) (Sigma-Aldrich) at a final concentration of 5 nM. For AR inhibition, cells were treated with either 2 μM bicalutamide or 5 μM enzalutamide (MedChem Express; Monmouth Junction, NJ). Compound doses were chosen to inhibit proliferation while sustaining at least 50% cell viability based on previous reports (enzalutamide^24^ and bicalutamide^25^). Cell viability was confirmed by MTT assays.

For epidermal growth factor (EGF) stimulation, cells were serum-starved (0.5% serum) for 24 hours, followed by stimulation with 10 ng/mL human recombinant EGF (Gibco, Thermo Fisher Scientific; Waltham, MA) for the times indicated in the figures.

For small interfering RNA (siRNA) experiments, cells were transfected with siGLO RISC-Free Control siRNA (D-001600-01-05) or SMARTpool ON-TARGETplus AR siRNA (L-003400-00-0005) with the Dharmacon DharmaFECT 2 siRNA transfection system (T-2002-02) (Dharmacon, GE Healthcare; Chicago, IL).

Clonogenic growth was measured using colony formation assays as previously described^26^. To assess anchorage-independent survival and proliferation, tumor spheroid formation was determined by culturing cells in low-adhesion 35 mm dishes in SpheroMax medium and supplement (PromoCell; Heidelberg Germany; C-28070) according to manufacturer’s protocols.

### Real-time quantitative PCR (RT-q-PCR)

Total RNA was isolated from cells with the Aurum Total RNA Mini Kit (Bio-Rad; Hercules, CA; 7326820) and reverse transcribed to cDNA with the iScript cDNA Synthesis Kit (Bio-Rad; 1708891). Primer sequences are listed in the supplementary Table S1. RT-q-PCR was performed using the SsoFast EvaGreen supermix (Bio-rad; 1725201) and the Bio-Rad CFX96 Real-Time PCR Detection System. Threshold cycle (C_T_) values were measured and relative abundance of target mRNA was calculated with the ΔC_T_ method relative to the housekeeping gene peptidylprolyl isomerase A (PPIA), while relative expression between treatments was determined with the ΔΔC_T_ method to calculate the fold difference (FD)^27^.

### Western blot

Total protein was quantified with Pierce Micro BCA Protein Assay Kit (Thermo Scientific; 23235). Equal amounts of total protein were loaded in each lane and separated by SDS-PAGE, electrophoretically transferred to polyvinylidene fluoride (PVDF) membranes, and probed with specific antibodies (antibody details are listed in Supplementary Table S2). Chemiluminescence was detected with Amersham ECL Prime Western Blotting Detection Reagents (GE Healthcare; RPN2109) and imaged on autoradiography films. Densitometric analysis of detected protein bands was performed with LI-COR Image Studio Digits v.3.1 (Lincoln, NE).

### CXCR7 promoter reporter assay

A CXCR7 promoter fragment (2,549 nucleotides [nt] upstream and 172 nt downstream of the transcription start site [TSS]) was PCR amplified from CalTech Human BAC Library D (CTD) clone 2207H15 plasmid DNA (ThermoFisher Scientific) with KpnI (5′) and XhoI (3′) restriction enzyme linker sequences (primer sequences listed in Supplementary Table S1). The promoter fragment was ligated into the pGL4.20[Luc2/Puro] promoterless luciferase reporter plasmid (Promega; Madison, WI; E6751), amplified, and isolated with PureYield Plasmid Maxiprep System (Promega; A2392). Cells were transfected with the plasmid using Lipofectamine 2000 (Thermo Fisher; 11668027), and selected with 2 μg/mL puromycin dihydrochloride (Sigma-Aldrich). Promoter luciferase activity was assayed using the Promega Luciferase Assay System (E1500), and detected by reading in the Promega GloMax 96 Microplate Luminometer.

### Chromatin Immunoprecipitation assay

Potential AR binding regions (AREs) were predicted in the CXCR7 promoter up to 6 kb upstream of the TSS using the online transcription factor prediction PROMO-ALGGEN algorithm^28, 29^. LNCaP cells were cultured in 10 cm dishes, androgen depleted for 48 hours, followed by continued depletion or stimulation with 5 nM R1881 for 24 hours. Cells were fixed for 10 minutes in 1% formaldehyde (Thermo Fisher, 16% [w/v] methanol free). Fixed cells were processed according to the Pierce Agarose ChIP Kit (Thermo Scientific; 26156) protocols, except for the following modifications. Following incubation in nuclear lysis buffer, brief sonication was performed to improve the yield of digested chromatin. ChIP DNA was recovered (pulldown antibody details are listed in Supplementary Table S2) and analyzed by RT-q-PCR (primer sets: Supplementary Table S1), relative to 10% input controls. Percent-input for samples incubated with AR antibody was compared relative to rabbit IgG controls to obtain fold-enrichment over background.

### CRISPR-Cas9 mediated genomic DNA mutation

CRISPR guide RNA (gRNA) sequence design and plasmid preparation were provided by Santa Cruz Biotechnologies (Dallas, TX), utilizing the Genome-Scale CRISPR Knock-Out (GeCKO) v2 library^30^. Three CXCR7-targeting CRISPR-Cas9-GFP plasmids were supplied as a combined pool: sc-403187A1 (CXCR7-gRNA 1), sc-403187A2 (CXCR7-gRNA 2), and sc-403187A3 (CXCR7-gRNA 3). The 20-nucleotide target sequences (Supplementary Table S1) along with the protospacer adjacent motif (PAM) sequences were confirmed to be specific to CXCR7 (Basic Local Alignment Search Tool [BLAST] search; National Center for Biotechnology Information, Bethesda MD) and further confirmed with the CasFinder algorithm^31^. Plasmids were transfected into cells using Lipofectamine 3000 Transfection Reagent (Invitrogen; L3000008). GFP positive cells were sorted using the BD FACSAria II (Augusta University flow cytometry core), and plated at single cell dilutions into 96-well plates for clonal expansion. Due to random insertion or deletion mutations (InDels) resulting from CRISPR-Cas9, clonal expansion was necessary to obtain homogeneous cell populations. Colonies were tested for CXCR7 knockout via western blot and Sanger sequencing of genomic DNA (GENEWIZ; South Plainfield, NJ).

### Senescence associated β-galactosidase activity assay

X-gal staining for senescence associated β-galactosidase (SA-gal) activity was carried out in CRISPR-Cas9-modified LNCaP and C4-2B cells. Fixation and staining of cells was carried out according to previously established protocols^32, 33^ (all reagents were obtained from Sigma-Aldrich). Cell lines expressing wild-type (WT) CXCR7 were treated with 10 μM H_2_O_2_ for 96 hours to initiate oxidative stress-induced cellular senescence as a positive control^34^.

### Proximity ligation assay

The Proximity Ligation Assay (PLA) (Duolink *In Situ* Red Starter Kit, Mouse/Rabbit; Sigma Aldrich; DUO92101) was performed according to the manufacturer’s protocol. Cell preparation and measurements were carried out as previously reported^13, 35^. Primary antibodies used for this assay are listed in Supplementary Table S2. Red-fluorescent spots indicate that target proteins co-localized within 40 nm of one another.

### Statistical analyses

All statistical analyses were carried out using Prism v.4.03 (GraphPad; La Jolla, CA). Significance between individual treatment groups and controls is calculated using unpaired, two-tailed Student’s t-tests. For siAR ± R1881 experiments, one-way ANOVA was used with the secondary Tukey’s multiple comparisons test to determine significance between treatment groups. Significance was considered achieved with p < 0.05 and is designated in the figures as: *p < 0.05, **p < 0.01, ***p < 0.001, and ns = no significance.

## Results

### CXCR7 expression is suppressed by androgen

**Figure 1:**
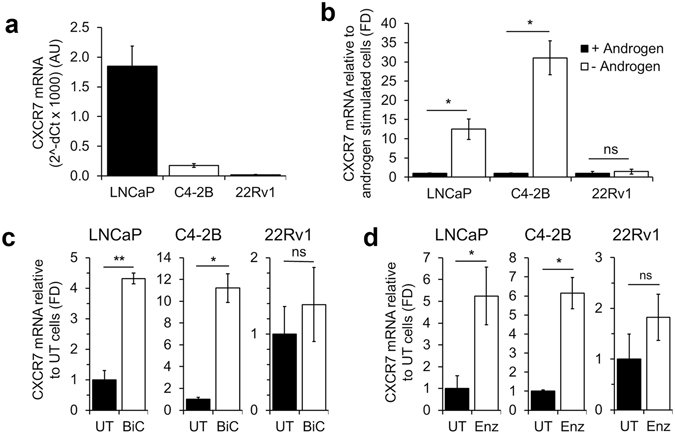
Androgen deprivation and AR inhibition result in increased CXCR7 mRNA transcription in androgen-responsive prostate cancer cells. (**a**) CXCR7 mRNA abundance in LNCaP, C4-2B, and 22Rv1 cell lines relative to PPIA. (**b**) CXCR7 mRNA transcription in androgen deprived cells relative to androgen stimulated cells. (**c**) CXCR7 mRNA in cells treated with 2 µM bicalutamide (BiC) relative to untreated (UT) cells. (**d**) CXCR7 mRNA in cells treated with 5 µM enzalutamide (Enz) relative to UT cells. All graphs shown include the means of 3 independent experiments, ± standard error of the mean (SEM). *p < 0.05, **p < 0.01, ns = no significance; unpaired, two-tailed Student’s t-test.

To determine if CXCR7 is upregulated during androgen deprivation, we investigated three prostate cancer cell lines with varying androgen responsiveness. We utilized androgen-dependent LNCaP cells; LNCaP-derived, castration resistant LNCaP-C4-2B (C4-2B) cells; and the CRPC cell line CRW-22Rv1 (22Rv1). Of these three lines under normal culture conditions (Fig. 1a), LNCaP cells had the highest expression of CXCR7 mRNA relative to the internal control housekeeping gene PPIA mRNA (1.85 ± 0.33 arbitrary units [AU]), followed by C4-2B cells (0.18 ± 0.03 AU). The 22Rv1 cell line yielded the lowest baseline CXCR7 mRNA transcription (0.02 ± 0.01 AU). Shown in Fig. 1b, CXCR7 mRNA significantly increased in both LNCaP and C4-2B cells in CDFBS supplemented media (- Androgen) relative to CDFBS + 5 nM R1881 (12.50 ± 2.65 fold in LNCaP, and 31.04 ± 4.42 fold in C4-2B). A slight but insignificant increase (1.45 ± 0.63 fold) was observed in 22Rv1 cells. To test if the suppression of CXCR7 is mediated by AR signaling, we utilized antiandrogen compounds (2 µM bicalutamide [BiC] or 5 µM enzalutamide [Enz]) to inhibit AR signaling under normal culture conditions. Cells were cultured in media supplemented with standard FBS, and treated with Enz, BiC, or left untreated (UT). Shown in Fig. 1c, BiC treatment resulted in increased CXCR7 mRNA transcription in LNCaP (4.32 ± 0.17 fold) and C4-2B (11.20 ± 1.31 fold) but not 22Rv1 (1.38 ± 0.48 fold) relative to UT cells. Figure 1d shows that Enz treatment of LNCaP cells relative to UT controls resulted in significantly increased CXCR7 mRNA (5.24 ± 1.32 fold). C4-2B responded with similar, significantly increased CXCR7 transcription (6.15 ± 0.82 fold), while Enz treatment of 22Rv1 cells only showed a mild increase (1.82 ± 0.46 fold). Together these results show that both inhibition of AR and androgen deprivation lead to significantly increased CXCR7 transcription in androgen-responsive prostate cancer cells.

### CXCR7-mediated mitogenic signaling increases during androgen deprivation

**Figure 2:**
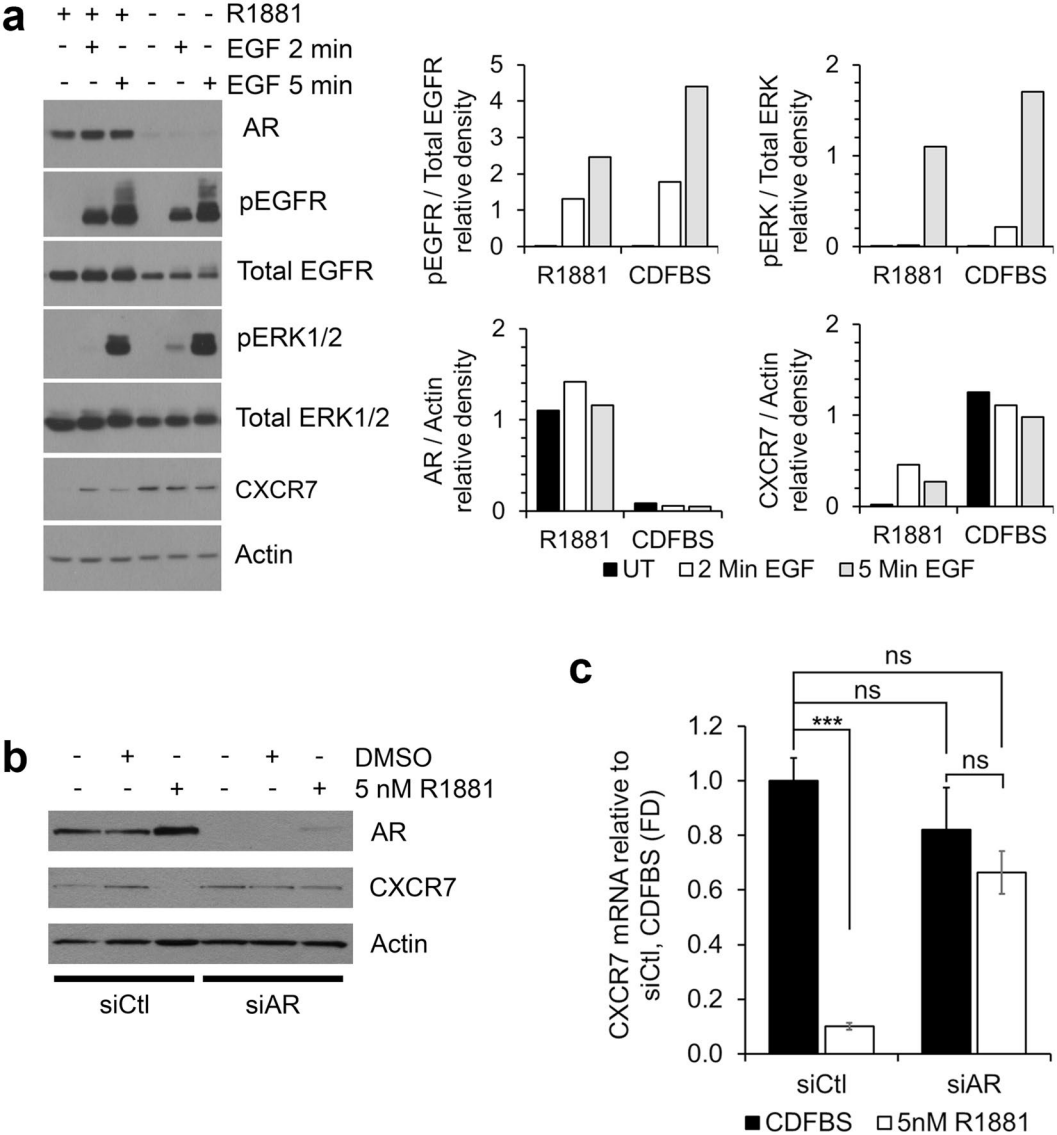
AR regulates CXCR7 expression and downstream EGFR-mediated mitogenic signaling. (**a**) Left: western blot panel of indicated proteins from LNCaP cells treated in the presence or absence of 5 nM R1881, followed by 2 or 5 minutes of EGF stimulation. Right: Densitometry analysis of phosphorylated protein relative to total; and AR or CXCR7 relative to actin. (**b**) Western blot panel of indicated proteins from LNCaP cells transfected with control siRNA (siCtl) or AR siRNA (siAR) treated with vehicle (DMSO) or 5 nM R1881. (**c**) CXCR7 mRNA expression in siCtl or siAR transfected cells with or without R1881 stimulation. n = 3, mean ± SEM; ***p < 0.001, ns = no significance; one-way ANOVA, secondary Tukey’s multiple comparisons test. The western blots shown in a and b are representative of three independent experiments with similar outcomes; densitometry is measured from the western blot panel shown.

CXCR7 has been shown to mediate transactivation of EGFR and downstream mitogenic signaling independent of SDF-1α^16^. We aimed to determine whether increased CXCR7 expression during androgen deprivation enhances EGF-mediated mitogenic signaling. LNCaP cells were treated in CDFBS supplemented media with or without R1881, followed by EGF stimulation. During androgen deprivation, CXCR7 protein was increased while AR protein was decreased (Fig. 2a, and replications shown in Supplementary Fig. S1). Densitometry analysis of the phosphorylation of EGFR at Y1110 (pEGFR) relative to total EGFR protein showed it was greater in androgen deprived cells at both 2 minutes (relative density = 1.79 [CDFBS], vs. 1.32 [R1881]) and 5 minutes (relative density = 4.40 [CDFBS], vs. 2.47 [R1881]) of EGF stimulation. Downstream ERK1/2 phosphorylation (pERK1/2) relative to total ERK1/2 was also increased in androgen deprived cells at 2 minutes (relative density = 0.22 [CDFBS], vs. 0.02 [R1881]) and 5 minutes (relative density = 1.70 [CDFBS], vs. 1.10 [R1881]) of EGF stimulation (Fig. 2a). These data reveal that CXCR7 overexpression during ADT enhances mitogenic signaling.

### AR is required for androgen-mediated CXCR7 suppression

We observed that androgen negatively regulates CXCR7 in prostate cancer cells. To show that AR is necessary for down-regulation of CXCR7, AR was transiently depleted in LNCaP cells and CXCR7 expression was measured in the presence or absence of androgen. Figure 2b shows western blot confirmation of AR depletion with siRNA. AR expression is maintained in the control siRNA (siCtl) transfected cells, and increases with androgen stimulation. In AR siRNA (siAR) transfected cells, AR protein was strongly depleted compared to siCtl transfected cells. CXCR7 protein decreased with androgen stimulation in the siCtl transfected cells; however, this effect is no longer observed with AR depletion. Furthermore, androgen stimulation resulted in a significant decrease in CXCR7 mRNA in siCtl transfected cells (Fig. 2c). Conversely, in AR depleted cells, androgen stimulation had no significant effect on CXCR7 mRNA transcription. These results reveal that the observed androgen-mediated suppression of CXCR7 is dependent on AR.

### AR directly suppresses CXCR7 transcription

**Figure 3:**
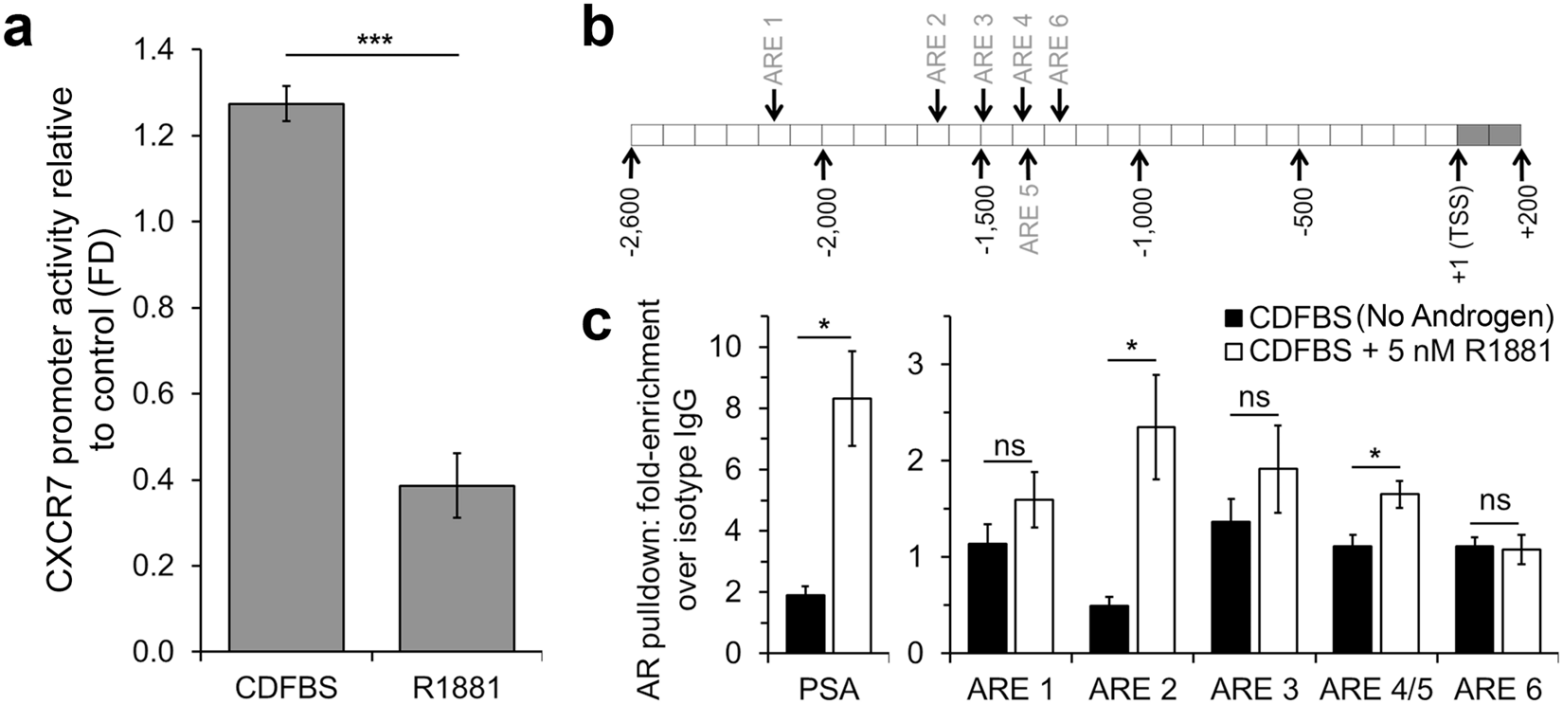
CXCR7 expression is directly regulated by AR binding to the CXCR7 promoter. (**a**) CXCR7 promoter-driven luciferase activity in C4-2B cells cultured in CDFBS ± R1881, relative to untreated control cells (n = 3, mean ± SEM). (**b**) Diagram of the cloned CXCR7 promoter region including the 6 putative AREs tested for AR binding. (**c**) ChIP assay for AR pulldown of promoter regions shown in B in LNCaP ± R1881. The PSA enhancer region served as a positive control for AR binding. n = 3, mean ± SEM; *p < 0.05, ***p < 0.001, ns = no significance; unpaired, two-tailed Student’s t-test.

We determined that androgen-mediated depletion of CXCR7 relies on AR in prostate cancer cells. However, it was unclear if this response was due to direct or indirect transcriptional regulation of CXCR7 by AR. A CXCR7-promoter-driven luciferase reporter plasmid was generated, containing 2,549 nt upstream and 172 nt downstream of the TSS, and stably transfected into C4-2B cells. Shown in Fig. 3a, stimulation with R1881 resulted in significantly reduced CXCR7-driven luciferase activity when compared to CDFBS alone.

We next investigated whether AR directly regulates CXCR7 transcription through interaction with one or more of the six putative AR binding elements (AREs) in the CXCR7 promoter sequence (depicted in the diagram in Fig. 3b). Chromatin immunoprecipitation (ChIP) assays were performed on the genomic CXCR7 promoter in LNCaP cells in the presence and absence of androgen. The promoter of prostate specific antigen (PSA) was used as a positive control for AR binding and showed a significant enrichment with androgen stimulation compared to androgen deprived cells (Fig. 3c-left). In the CXCR7 promoter (Fig. 3c-right), there was a significant increase in AR binding at the predicted ARE 2 region (R1881: 2.35 ± 0.54 vs. CDFBS: 0.49 ± 0.09) as well as the region containing the closely spaced (7 nt separation) AREs 4 and 5 (ARE 4/5) (R1881: 1.65 ± 0.14 vs. CDFBS: 1.11 ± 0.12). Androgen stimulation also increased AR enrichment of the ARE 1 and ARE 3 regions, but did not reach significance. There was no change in enrichment at the ARE 6 region. These results reveal that AR directly binds to the CXCR7 promoter at two predicted ARE-containing regions, suggesting AR directly regulates CXCR7 transcription in the presence of androgen.

### CRISPR-Cas9 knockout of CXCR7 in C4-2B cells strongly reduces cell proliferation

**Figure 4:**
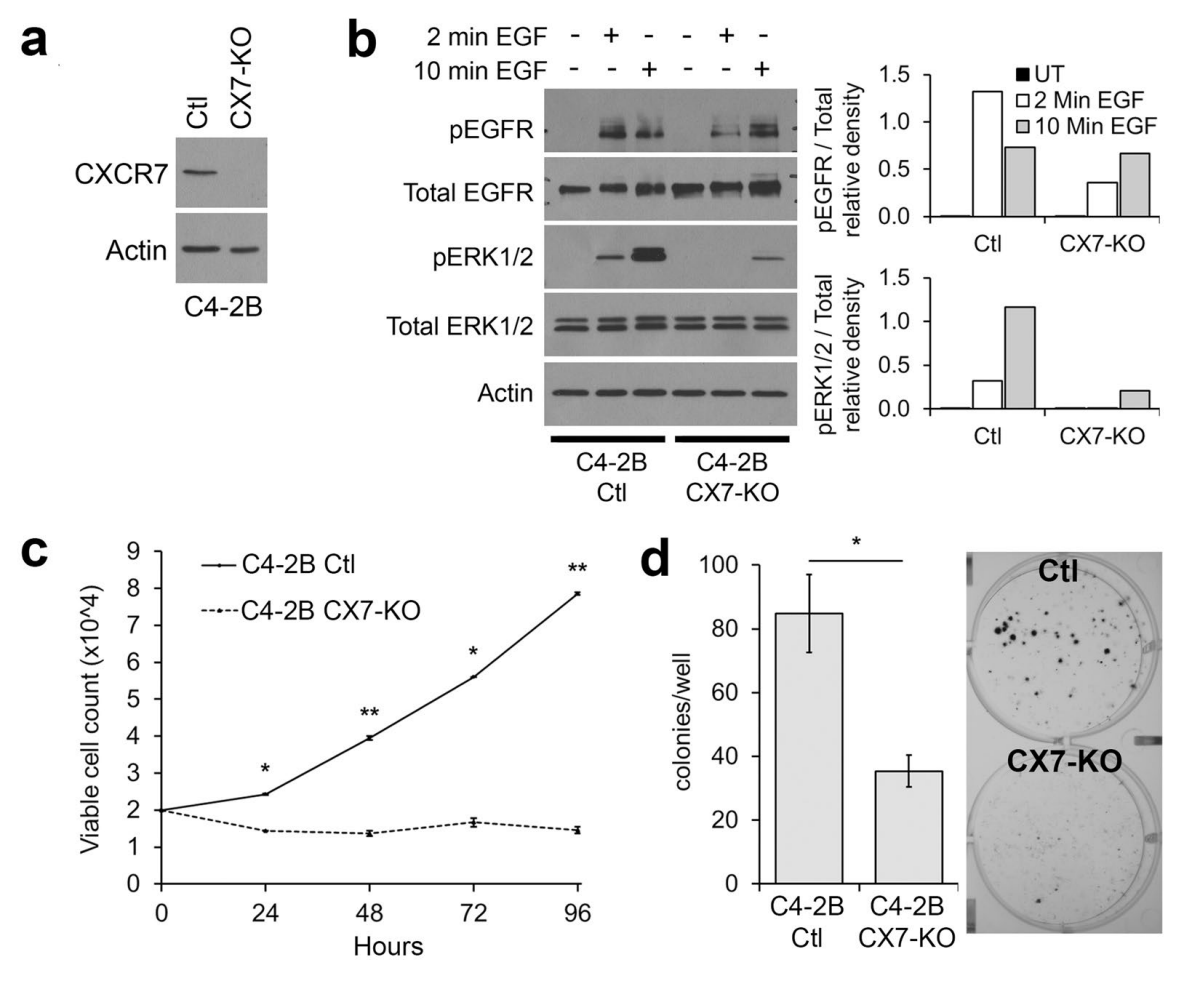
CRISPR-Cas9 deletion of CXCR7 inhibits cell proliferation and suppresses mitogenic signaling. (**a**) Western blot confirmation of CXCR7 protein knockout in C4-2B cells. (**b**) Western blots of EGFR and ERK1/2 activation in Ctl and CX7-KO cells, and densitometry showing relative phosphorylated protein to total protein. (**c**) Viable cell count for Ctl and CX7-KO cells (n = 2, mean ± SEM). (**d**) Left: Clonogenic survival assay results displaying a mean count of colonies obtained per well for each cell line. Right: representative image of Ctl and CX7-KO established colonies (n = 3, mean ± SEM). *p < 0.05, **p < 0.01; unpaired, two-tailed Student’s t-test. The western blots are representative of two independent experiments with similar outcomes; densitometry is measured from the western blot panel shown.

We have shown that CXCR7 is directly repressed by AR, and that CXCR7 overexpression facilitates androgen-independent mitogenic signaling. Our next aim was to determine if CXCR7 is essential for this alternative signaling pathway to maintain androgen-independent cancer cell survival. Because previous work using stable short hairpin RNA (shRNA) expression was inefficient to maintain long-term CXCR7 depletion (data not shown), we utilized CRISPR-Cas9 to completely abrogate CXCR7 expression. Figure 4a shows the western blot confirmation of CXCR7 protein knockout in one C4-2B clone (CX7-KO) relative to control, non-specific CRISPR gRNA transfected C4-2B cells (Ctl). To investigate whether CX7-KO cells show differential sensitivity to the EGF stimulated mitogenic response, Ctl and CX7-KO cells were stimulated for 2 or 10 minutes with EGF after 24 h culture in low serum medium. Shown in Fig. 4b (repetition shown in Supplementary Fig. S2), in the CX7-KO cell line, pEGFR relative to total EGFR was reduced when compared to Ctl cells after 2 minutes of EGF stimulation (relative density = 1.32 [Ctl] vs. 0.35 [CX7-KO]). After 10 minutes of EGF stimulation, the pEGFR/total EGFR ratio in Ctl cells declined but remained greater than in CX7-KO cells (relative density = 0.73 [Ctl] vs. 0.66 [CX7-KO]). Downstream pERK1/2 relative to total ERK1/2 was also reduced in CX7-KO compared to Ctl cells at 2 minutes (relative density = 0.32 [Ctl] vs. 0.01 [CX7-KO]), and 10 minutes of EGF stimulation (relative density = 1.16 [Ctl] vs. 0.21 [CX7-KO]). Viable cell counts over 96 hours (Fig. 4c) showed the Ctl cells proliferate significantly faster than the CXCR7-KO cells, with the final count at 7.85 × 10^4^ ± 0.37 × 10^3^ viable cells in the Ctl line and a decrease to 1.46 × 10^4^ ± 0.92 × 10^3^ viable cells in the CX7-KO line. Fig. 4d shows a representative image of a colony formation assay as well as a count of colonies formed. Smaller and significantly fewer colonies were obtained from CX7-KO cells (35.3 ± 5.0 colonies per well) compared to Ctl cells (84.8 ± 12.3 colonies per well). After 2 passages, cells from this CX7-KO clone failed to proliferate and could not be recovered in culture.

### CRISPR-Cas9 frameshift mutations of CXCR7 generate a senescent phenotype

**Figure 5:**
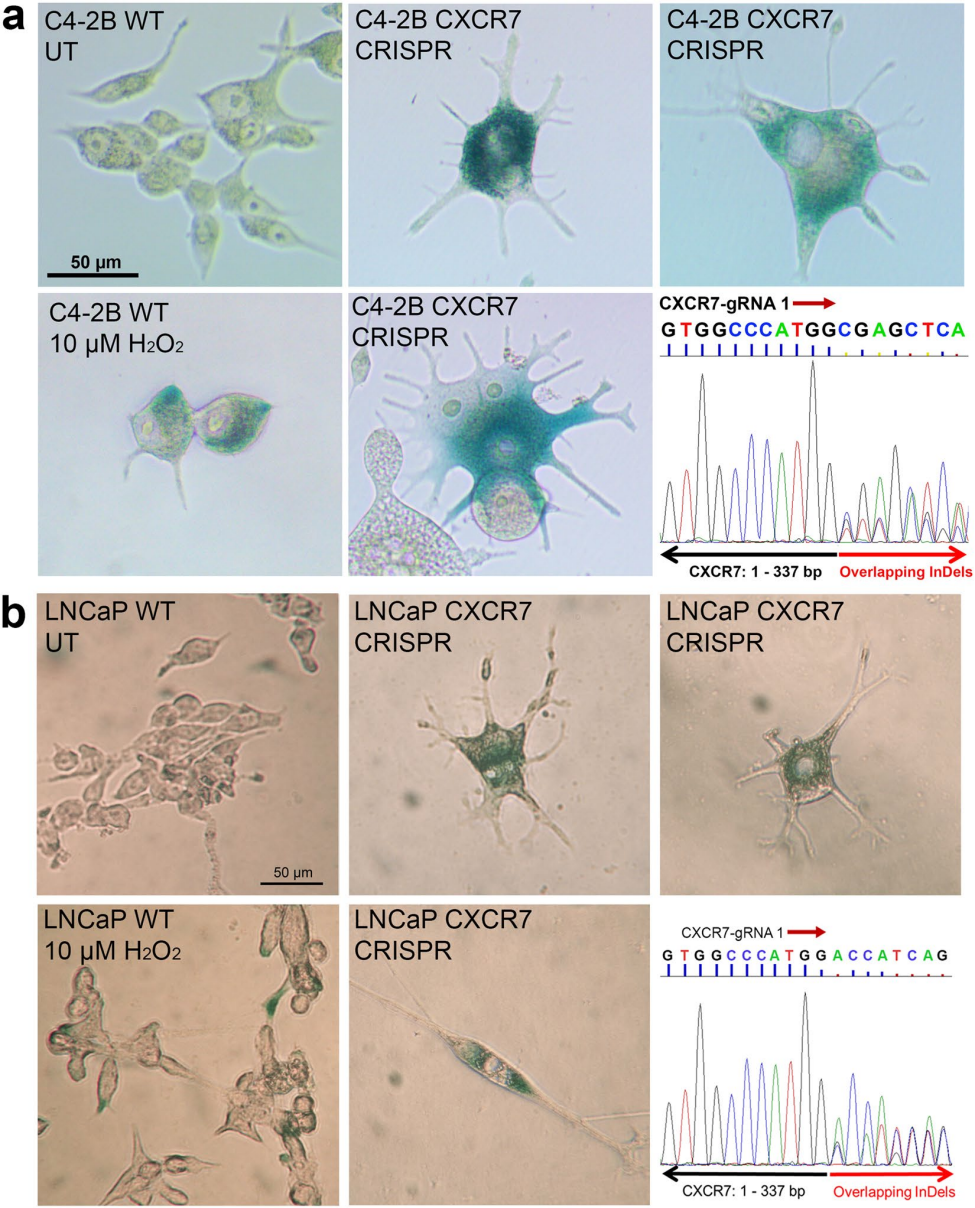
CRISPR-Cas9-mediated frameshift mutations of CXCR7 resulted in the onset of cellular senescence. (**a**) X-gal assay for SA-β-galactosidase (SA-gal) activity in C4-2B CXCR7 frameshift mutant cells. Positive SA-gal expression is indicated by dark blue coloration to the cells. Upper-left panel: C4-2B cells with wild-type (WT) CXCR7 expression untreated (UT) (negative control), Lower-left panel: WT C4-2B cells treated with 10 µM H_2_O_2_ (positive control). Upper-middle, lower middle, and upper-right panels: representative images in CXCR7-CRISPR-mutated cells. Lower-right panel: Sanger sequencing chromatogram of the CXCR7-gRNA target site illustrating overlapping unique CXCR7 InDel mutations. (**b**) X-gal assay in LNCaP CXCR7 frameshift mutant cells and controls (UT or H_2_O_2_ treated).

We repeated CRISPR-Cas9 editing in C4-2B cells to obtain new CXCR7-KO colonies. However, several of these colonies exhibited the same proliferative arrest observed previously. Colonies were passaged once, with 10% of the cells harvested for genomic DNA sequencing. Remaining cells were returned to culture. After one month in culture, no further proliferation could be observed, and all cells had developed a broad, flattened morphology with numerous long protrusions. In these colonies we performed staining for senescence-associated β-galactosidase (SA-gal) activity. Figure 5a shows the results of these tests in a representative colony of CRISPR modified C4-2B cells. C4-2B cells expressing wild-type (WT) CXCR7 were stained for SA-gal activity as a negative control (no dark blue staining) (Fig. 5a, upper left panel). WT C4-2B cells were also treated with 10 µM H_2_O_2_ to induce oxidative damage-mediated cellular senescence (positive control). Figure 5a, lower left panel shows a representative image of these H_2_O_2_ treated cells staining blue, indicating SA-gal activity. In proliferation-arrested CRISPR-Cas9 mutated C4-2B colonies, strong blue staining was observed in nearly all cells (Fig. 5a: representative images in upper-middle, lower-middle, and upper-right panels) indicating cellular senescence. The lower-right panel of Fig. 5a shows the Sanger sequencing chromatogram of CXCR7 genomic DNA at the CXCR7-gRNA 1 target site of this clone. The overlapping InDel mutations for CXCR7 indicate that this colony had frameshift mutations in the CXCR7 gene caused by CRISPR-Cas9.

In a parallel experiment, transfection of LNCaP cells with CXCR7-targeting CRISPR-Cas9 gRNA resulted in one normally proliferating colony (three weeks to establish, results discussed in the next section) and two slow-growing colonies (two months to establish). Cultures were passaged once, with 10% harvested for genomic DNA sequencing. Remaining cells were returned to culture. As with C4-2B cells, after passaging cells once, no proliferation could be observed in the two previously slow-growing LNCaP colonies, and the cells developed a broad, flattened morphology. These cells were tested for SA-gal activity. The left two panels of Fig. 5b show WT-CXCR7 LNCaP cells ± 10 µM H_2_O_2_. H_2_O_2_ treated LNCaP cells showed strong blue staining indicating SA-gal expression which was not observed in UT control cells. The upper-middle, lower-middle, and upper-right panels in Fig. 5b are representative images of the non-proliferating CRISPR-Cas9 LNCaP cells, exhibiting a broad, flattened morphology, and dark blue X-gal staining, indicative of SA-gal expression, which are all hallmarks of cellular senescence^32, 34^. Genomic DNA sequencing results for the senesced clones (representative chromatogram in the lower-right panel of Fig. 5b), shows overlapping sequences beginning 3 nucleotides downstream of the Cas9 cleavage site targeted by the CXCR7-gRNA 1 construct. The overlapping signal is indicative of unique InDel mutations. These results, together with the observations in C4-2B cells suggest that loss of CXCR7 leads to severely inhibited proliferation and eventual cellular senescence.

### Functional characterization of a LNCaP clone with a CXCR7 inframe mutation

**Figure 6:**
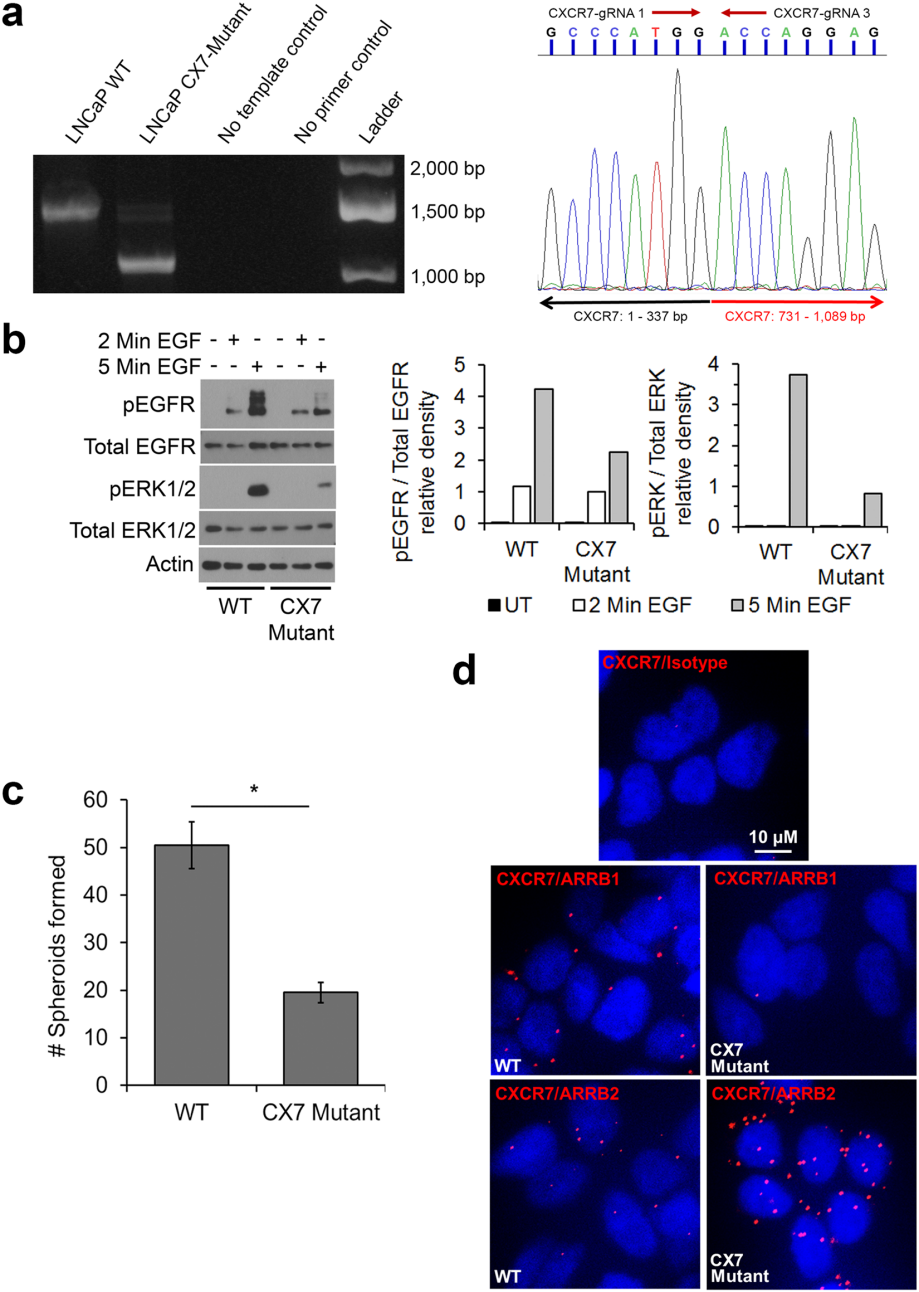
CRISPR-Cas9-generated CXCR7 inframe mutation in LNCaP cells disrupts membrane protein interactions. (**a**) Left: PCR of CXCR7 genomic DNA in unmodified cells (WT) and CRISPR-edited LNCaP cells revealing a 394 nt deletion event (CX7-mutant). Right: Sanger sequencing chromatogram of the DNA sequence illustrating the contiguous sequence, Black arrow represents the expected 5′ CXCR7 sequence and the red arrow shows the 3′ CXCR7 sequence around the site of deletion event. (**b**) EGFR and ERK phosphorylation in WT and CX7-mutant cells with densitometry of phosphorylated protein relative to total. (**c**) Count of second generation spheroids formed per plate from 10,000 seeded WT and CX7-mutant cells (n = 2, mean ± SD; *p < 0.05; unpaired, two-tailed Student’s t-test. (**d**) PLA staining for CXCR7 protein interaction with ARRB1, ARRB2, or isotype control in the WT and CX7-mutant cell lines. Nuclei are stained with DAPI (blue); red spots indicate that the two target proteins are co-localized within 40 nm of one another. The western blots are representative of three independent experiments with similar outcomes; densitometry is measured from the western blot panel shown.

One normally proliferating CRISPR-Cas9 modified LNCaP clone was obtained which did not show the same senescent phenotypes. PCR amplification of both the CXCR7 genomic DNA and the mRNA-derived cDNA both indicated a 394 nt deletion between two CRISPR gRNA target sites (CXCR7-gRNA 1 and CXCR7-gRNA 3) (corresponding to amino acids 114 to 243 of the CXCR7 protein) (Fig. 6a-left). A faint upper band is apparent in the CXCR7-mutant DNA PCR lane, but sequencing of this fragment indicated a similar alternative InDel sequence overlap to that observed in the slow-growing colonies (data not shown). Sanger sequencing of the shorter PCR band revealed that the coding sequence for both N and C terminal ends of the CXCR7 protein were maintained (Fig. 6a-right). Unlike the frameshift mutations in the previously described LNCaP clones, this clone did not exhibit signs of cellular senescence. To determine if the deletion of the internal CXCR7 sequence was necessary for CXCR7-mediated mitogenic signaling, we measured EGFR and ERK1/2 phosphorylation. The western blot densitometry (figure 6b-right) shows that pEGFR relative to total EGFR is greater in WT cells than in CXCR7-mutants at 2 minutes (relative density = 1.18 [WT] vs. 1.00 [CX7-mutant]), and 5 minutes (relative density = 4.21 [WT] vs. 2.24 [CX7-mutant]) after EGF stimulation. pERK1/2 relative to total ERK1/2 is not detectable in either line until 5 minutes of EGF stimulation (relative density = 3.73 [WT] vs. 0.81 [CX7-mutant]) (Repetitions are shown in Supplementary Fig. S3).

We also investigated whether this mutation had an effect on the migration or metastatic phenotypes in LNCaP. A previous study determined that cell motility driven by SDF-1α is dependent on the CXCR4, CXCR7, AR regulatory axis^36^. We analyzed whether the CXCR7 mutant exhibited any disruption in cell motility compared to WT LNCaP cells. Transwell migration assays along an SDF-1α gradient resulted in no significant change in SDF1-α-stimulated migration in the CXCR7-mutant compared to WT (Supplementary Fig. S4). While the migratory potential is one aspect of metastatic potential, another necessary mechanism is anchorage-independent cell survival during transport through circulation^37^. Anchorage-independent sphere formation is an indicator of the metastatic potential of a cancer cell line^38^. The sphere forming potential of the CXCR7-mutant is significantly suppressed compared to WT cells (50.5 ± 4.95 spheroids [WT] vs. 19.5 ± 2.12 spheroids [CX7-mutant]) (Fig. 6c).

Previous work in our lab showed that CXCR7-mediated EGFR phosphorylation, downstream mitogenic signaling, and tumor cell proliferation are inhibited by β-arrestin 2 (ARRB2)^35^. Alternatively, others have shown β-arrestin 1 (ARRB1) mediates CXCR7 transactivation of EGFR^39, 40^. To determine if the mutation altered CXCR7-ARRB1 or CXCR7-ARRB2 interactions leading to reduced EGFR transactivation, we performed PLAs to probe for co-localization of CXCR7 and either ARRB1 or ARRB2 (Fig. 6d). We observed substantially reduced CXCR7-ARRB1 and increased CXCR7-ARRB2 interactions in the CXCR7-mutant LNCaP cell line compared to WT CXCR7 LNCaP cells. These data reveal that the lost protein region containing amino acids 114 to 243 altered protein-protein interactions necessary for the observed ARRB-EGFR-CXCR7 regulatory axis, providing a potential explanation for disrupted EGF-mediated mitogenic signaling.

Together our data show that functional CXCR7 is necessary for prostate cancer cell proliferation even in the presence of androgen. Complete CXCR7 ablation resulted in compromised mitogenic signaling and onset of cellular senescence.

## Discussion

**Figure 7:**
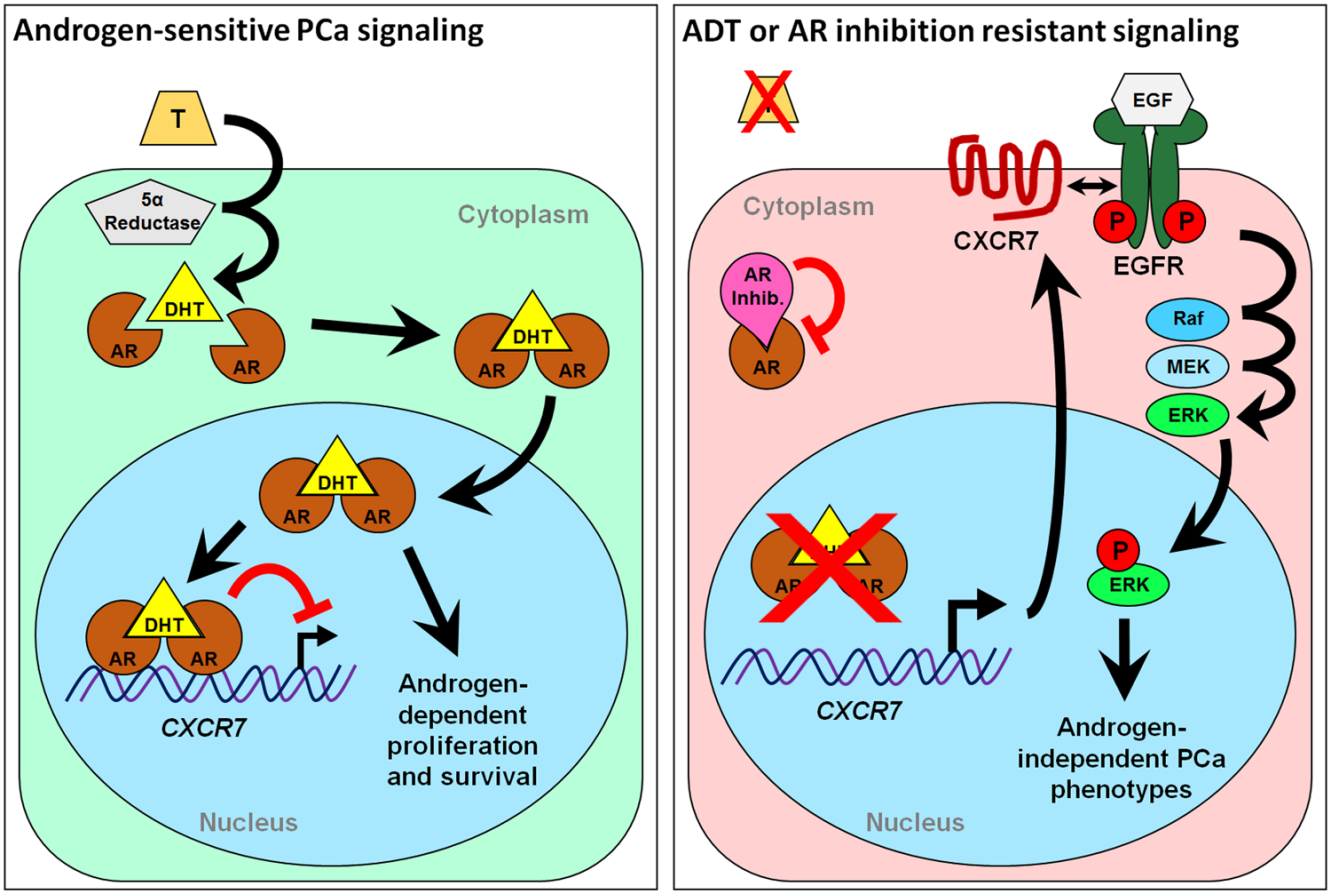
Illustration of the proposed prostate cancer cell signaling programs with or without normal androgen stimulation. Left: normal androgen signaling (testosterone [T] converted to dihydrotestosterone [DHT] and AR activation) suppresses CXCR7 expression while driving androgen-dependent proliferation mechanisms. Right: CXCR7 repression is removed during androgen deprivation therapy (ADT) or with chemical AR inhibition, facilitating EGF-mediated, androgen-independent cell survival and proliferation, and mechanisms of therapeutic resistance.

This study provides compelling evidence for the role of CXCR7 in prostate cancer survival following androgen ablation. When AR signaling is lost during ADT, increased CXCR7 expression drives EGF-induced mitogenic signaling as one mechanism that potentially facilitates prostate cancer cell survival. We show that both AR inhibition, and depletion of androgens lead to increased CXCR7 expression (Figs 1 and 2). We also determined that the increased CXCR7 expression in prostate cancer cells during ADT enhances EGF-induced EGFR and ERK1/2 activation (Fig. 2). For the first time we revealed that AR directly interacts with the CXCR7 promoter, suppressing transcription; a regulatory mechanism that is released during androgen deprivation (Fig. 3). This study also reveals a vital link between CXCR7 and proliferation of prostate cancer cells. CRISPR-Cas9-mediated deletion of CXCR7 in C4-2B cells repressed mitogenic signaling, cell proliferation, and colony formation potential (Fig. 4). We also determined in both C4-2B and LNCaP cells that frameshift mutations of CXCR7 result in the onset of cellular senescence (Fig. 5). As illustrated in Fig. 7, our results support a regulatory axis whereby CXCR7 is upregulated in the absence of AR signaling, which drives alternative EGF-mediated cell survival signaling, leading to androgen-independent prostate cancer progression. Furthermore, loss of CXCR7 in these cell lines leads to strongly reduced cell proliferation and clonogenic survival and promotes cellular senescence.

Factors which are dysregulated during ADT that support early androgen-independent cell survival or proliferation are vital targets for preventing disease progression. In this study we have focused on the atypical chemokine receptor CXCR7 as it was shown in previous work in our lab to be associated with cancer cell proliferation and survival^13, 16^. We have demonstrated that increased CXCR7 in the absence of AR signaling potentiates EGFR-mediated mitogenic signaling in prostate cancer cells (Fig. 2). Previous studies have determined that EGFR activation and mitogenic signaling cascades are involved in androgen-independent prostate cancer tumor cell survival and proliferation, and are important factors in the development of CRPC following androgen ablation^20, 21^. Interestingly, in androgen-sensitive prostate cancer cell lines deprived of androgen, we observed a decrease in total EGFR protein expression. Consistent with our results, it was previously reported that AR has a regulatory role over EGFR mRNA and protein expression whereby androgen deprivation resulted in decreased EGFR expression compared to androgen stimulated LNCaP cells^41^. However, the substantial depletion of total EGFR that we observed in our study did not result in any loss in phosphorylation. As such, the increase in CXCR7 during androgen depletion is capable of at least maintaining the same EGFR phosphorylation potential as observed under normal culture conditions. We have shown here in this work that CXCR7 can counter the depletion of EGFR and maintain normal mitogenic signaling during ADT, further supporting androgen-independent cell survival. The potentiation of androgen-independent mitogenic signaling by overexpressed CXCR7 reveals a mechanism supporting hormone refractory phenotypes leading to CRPC.

Provided that CXCR7 confers survival and growth advantages to prostate cancer cells, it is surprising that androgen deprivation resistant cell lines (C4-2B and 22Rv1) exhibit lower CXCR7 expression than androgen-dependent lines (LNCaP) as shown in Fig. 1a. This phenomenon may be explained by the mechanisms driving androgen independence in these cell lines. C4-2B cells exhibit increased expression of AR, allowing these cells to respond to greatly reduced circulating androgen levels compared to what is required by LNCaP cells to proliferate^42^. Overexpression of AR in C4-2B cells therefore results in a profound decrease in CXCR7 compared to LNCaP cells, as we have observed (Fig. 1a). This regulatory axis is further confirmed with our comparison to the androgen-independent cell line, 22Rv1. These cells proliferate completely independent of androgens by expressing constitutively active, ligand-independent AR splice variants (AR-Vs) which are capable of maintaining AR signaling regardless of the presence of androgens^43^. Because of the constitutive AR-V signaling in those cell lines, CXCR7 expression is exceedingly low, and there is no significant increase with either androgen depletion or AR inhibition. While AR signaling remains the primary factor driving prostate cancer, even in the progression to CRPC^44^, our results demonstrate that expression of CXCR7 is essential for prostate cancer cell survival and a critical factor during the transition to castration resistance.

We utilized CRISPR-Cas9 to eliminate CXCR7 expression in these prostate cancer cell lines. Interestingly, our results revealed CXCR7 expression is necessary for prostate cancer proliferation even under normal culture conditions (in the presence of androgen). Few colonies could be established from the CXCR7 CRISPR-gRNA transfected cells, and of those, clones exhibiting complete ablation of CXCR7 displayed cell proliferation arrest and onset of cellular senescence compared to control CRISPR-Cas9 transfected cells (Fig. 5). This is consistent with previous observations utilizing RNAi methods to deplete CXCR7 in prostate cancer cell lines under normal culture conditions (in the presence of androgen) which resulted in significantly reduced cell proliferation and inhibited cell cycle progression^16^. CRISPR-Cas9-mediated abrogation of CXCR7 likely enhanced these phenotypes since siRNA depletion could not completely eliminate CXCR7 expression. These observations support the critical role of CXCR7 in maintaining cell proliferation even under a normal androgen signaling state.

Surprisingly, in the Sanger sequencing chromatograms obtained from our CRISPR-Cas9 edited clones (Fig. 5), we observe up to three overlapping sequence traces. A single cell clone with two CXCR7 alleles should only exhibit up to two overlapping sequences with unique InDel events. One possible explanation is that residual CRISPR-Cas9 nuclease activity during the first few mitotic divisions resulted in a second CRISPR-mediated mutation to CXCR7 in one daughter cell. The resulting cell population would thereby exhibit three unique InDel mutations instead of the expected two. However, neither of three potential sequences represent wild-type CXCR7.

LNCaP cells exhibiting frameshift mutations in the CXCR7 protein coding region required nearly two months to establish colonies and, after one passage, exhibited a complete loss of cell proliferation and eventually senesced. Interestingly, the one colony expressing a inframe-mutation that maintained both the N- and C-terminal sequence around the deletion event continued to proliferate. However, analysis of this clone reveals substantial reduction in EGFR transactivation and mitogenic signaling. Reduction of EGFR transactivation in this clone may be explained through differential interactions of CXCR7 with ARRBs. Previous work in our lab has shown that ARRB2-CXCR7 interaction inhibits EGFR-Y1110 phosphorylation and downstream ERK1/2 activation in prostate cancer cells^35^. Alternatively, ARRB1 has also been shown to enhance the activation of EGFR in colorectal cancers leading to increased mitogenic signaling^39^. Furthermore, ARRB1 mediates CXCR7 ligand-dependent activation of ERK signaling in glioma^40^ as well as migration and mitogenic signaling mediated by CXCR4-CXCR7 heterodimers^45^. We performed PLA between CXCR7 and ARRB1 or ARRB2 to determine if the CXCR7 mutation alters these protein-protein interactions. As shown in Fig. 6d, mutant CXCR7 exhibits increased ARRB2 and reduced ARRB1 interactions compared to WT CXCR7. These results are consistent with the reduced EGFR and ERK1/2 signaling as shown in the literature^35, 39, 40, 45^.

## Conclusions

The data presented in this study reveal the vital role of CXCR7 in promoting survival and proliferation of prostate cancer tumor cells by potentiating alternative, androgen-independent mitogenic signaling pathways. This study demonstrates that inhibition of AR signaling results in increased CXCR7 expression and associated downstream mitogenic signaling. Together, our data demonstrate CXCR7 as a potential target for adjuvant therapy in conjunction with ADT to prevent progression to CRPC.

## Electronic supplementary material

Supplementary Information

## References

[1] Torre LA (2015). Global cancer statistics, 2012. CA: a cancer journal for clinicians.

[2] Gjertson CK (2007). Local control and long-term disease-free survival for stage D1 (T2-T4N1-N2M0) prostate cancer after radical prostatectomy in the PSA era. Urology.

[3] Ischia J, Gleave M (2013). Radical prostatectomy in high-risk prostate cancer. International journal of urology: official journal of the Japanese Urological Association.

[4] Sailer SL (2006). Radiation therapy for prostate cancer: external beam, brachytherapy, and salvage. North Carolina medical journal.

[5] Harris WP, Mostaghel EA, Nelson PS, Montgomery B (2009). Androgen deprivation therapy: progress in understanding mechanisms of resistance and optimizing androgen depletion. Nature clinical practice. Urology.

[6] Zlotnik A, Morales J, Hedrick JA (1999). Recent advances in chemokines and chemokine receptors. Critical reviews in immunology.

[7] Salazar N, Castellan M, Shirodkar SS, Lokeshwar BL (2013). Chemokines and chemokine receptors as promoters of prostate cancer growth and progression. Critical reviews in eukaryotic gene expression.

[8] Pierce KL, Premont RT, Lefkowitz RJ (2002). Seven-transmembrane receptors. Nature reviews. Molecular cell biology.

[9] Araki S (2007). Interleukin-8 is a molecular determinant of androgen independence and progression in prostate cancer. Cancer research.

[10] Maksym RB (2009). The role of stromal-derived factor-1–CXCR7 axis in development and cancer. European journal of pharmacology.

[11] Wang H (2015). CXCR7 functions in colon cancer cell survival and migration. Experimental and therapeutic medicine.

[12] Miao Z (2007). CXCR7 (RDC1) promotes breast and lung tumor growth *in vivo* and is expressed on tumor-associated vasculature. Proceedings of the National Academy of Sciences of the United States of America.

[13] Salazar N (2014). The chemokine receptor CXCR7 interacts with EGFR to promote breast cancer cell proliferation. Molecular cancer.

[14] Lin L (2014). CXCR7 stimulates MAPK signaling to regulate hepatocellular carcinoma progression. Cell death & disease.

[15] Wang J (2008). The role of CXCR7/RDC1 as a chemokine receptor for CXCL12/SDF-1 in prostate cancer. The Journal of biological chemistry.

[16] Singh RK, Lokeshwar BL (2011). The IL-8-regulated chemokine receptor CXCR7 stimulates EGFR signaling to promote prostate cancer growth. Cancer research.

[17] Yang D (2015). Expression of chemokine receptor CXCR7 in colorectal carcinoma and its prognostic significance. International journal of clinical and experimental pathology.

[18] Iwakiri S (2009). Higher expression of chemokine receptor CXCR7 is linked to early and metastatic recurrence in pathological stage I nonsmall cell lung cancer. Cancer.

[19] Katzenwadel A, Wolf P (2015). Androgen deprivation of prostate cancer: Leading to a therapeutic dead end. Cancer letters.

[20] Lorenzo GD, Bianco R, Tortora G, Ciardiello F (2003). Involvement of growth factor receptors of the epidermal growth factor receptor family in prostate cancer development and progression to androgen independence. Clinical prostate cancer.

[21] Saraon P, Jarvi K, Diamandis EP (2011). Molecular alterations during progression of prostate cancer to androgen independence. Clinical chemistry.

[22] Ran FA (2013). Genome engineering using the CRISPR-Cas9 system. Nature protocols.

[23] Gleave M, Hsieh JT, Gao CA, von Eschenbach AC, Chung LW (1991). Acceleration of human prostate cancer growth *in vivo* by factors produced by prostate and bone fibroblasts. Cancer research.

[24] Liu C (2014). Inhibition of constitutively active Stat3 reverses enzalutamide resistance in LNCaP derivative prostate cancer cells. The Prostate.

[25] Kawata H (2010). Prolonged treatment with bicalutamide induces androgen receptor overexpression and androgen hypersensitivity. The Prostate.

[26] Rafehi H (2011). Clonogenic assay: adherent cells. Journal of visualized experiments: JoVE.

[27] Schmittgen TD, Livak KJ (2008). Analyzing real-time PCR data by the comparative C(T) method. Nature protocols.

[28] Messeguer X (2002). PROMO: detection of known transcription regulatory elements using species-tailored searches. Bioinformatics.

[29] Farre D (2003). Identification of patterns in biological sequences at the ALGGEN server: PROMO and MALGEN. Nucleic acids research.

[30] Shalem O (2014). Genome-scale CRISPR-Cas9 knockout screening in human cells. Science.

[31] Aach, J., Mali, P. & Church, G. M. CasFinder: Flexible algorithm for identifying specific Cas9 targets in genomes. *bioRxiv*, doi:10.1101/005074 (2014).

[32] Debacq-Chainiaux F, Erusalimsky JD, Campisi J, Toussaint O (2009). Protocols to detect senescence-associated beta-galactosidase (SA-betagal) activity, a biomarker of senescent cells in culture and *in vivo*. Nature protocols.

[33] Itahana K, Itahana Y, Dimri GP (2013). Colorimetric detection of senescence-associated beta galactosidase. Methods in molecular biology.

[34] Kiyoshima T (2012). Oxidative stress caused by a low concentration of hydrogen peroxide induces senescence-like changes in mouse gingival fibroblasts. International journal of molecular medicine.

[35] Kallifatidis G (2016). beta-Arrestin-2 Counters CXCR7-Mediated EGFR Transactivation and Proliferation. Molecular cancer research: MCR.

[36] Hsiao JJ (2015). Androgen receptor and chemokine receptors 4 and 7 form a signaling axis to regulate CXCL12-dependent cellular motility. BMC cancer.

[37] Thiery JP (2002). Epithelial-mesenchymal transitions in tumour progression. Nature reviews. Cancer.

[38] Denes V (2015). Metastasis blood test by flow cytometry: *in vivo* cancer spheroids and the role of hypoxia. International journal of cancer.

[39] Buchanan FG (2006). Role of beta-arrestin 1 in the metastatic progression of colorectal cancer. Proceedings of the National Academy of Sciences of the United States of America.

[40] Odemis V (2012). The presumed atypical chemokine receptor CXCR7 signals through G(i/o) proteins in primary rodent astrocytes and human glioma cells. Glia.

[41] Pignon JC (2009). Androgen receptor controls EGFR and ERBB2 gene expression at different levels in prostate cancer cell lines. Cancer research.

[42] Gregory CW, Johnson RT, Mohler JL, French FS, Wilson EM (2001). Androgen receptor stabilization in recurrent prostate cancer is associated with hypersensitivity to low androgen. Cancer research.

[43] Dehm SM, Tindall DJ (2011). Alternatively spliced androgen receptor variants. Endocrine-related cancer.

[44] Tsao CK, Galsky MD, Small AC, Yee T, Oh WK (2012). Targeting the androgen receptor signalling axis in castration-resistant prostate cancer (CRPC). BJU international.

[45] Decaillot FM (2011). CXCR7/CXCR4 heterodimer constitutively recruits beta-arrestin to enhance cell migration. The Journal of biological chemistry.

