# Supplementary material for: Inhibition of androgen receptor promotes CXC-chemokine receptor 7-mediated prostate cancer cell survival
Source: Sci Rep. 2017 Jun 8;7:3058. doi: 10.1038/s41598-017-02918-3 (PMC5465216; doi:10.1038/s41598-017-02918-3)
Supplement: Supplementary file 1 — Supplementary Information [file 41598_2017_2918_MOESM1_ESM.pdf]

Inhibition of androgen receptor promotes CXC-chemokine receptor 7-mediated prostate cancer cell survival

James J. Hoy, Georgios Kallifatidis, Diandra K. Smith, and Bal L. Lokeshwar

Supplementary Table S1: primer sets and CRISPR gRNA sequences

**RT-q-PCR primers**	
CXCR7 Forward	5'-GCACAGCACAGCCAGGAAGG-3’
CXCR7 Reverse	5’-TCGTTCTGAGGCGGGCAATC-3’
PPIA Forward	5’-CTTGGGCCGCGTCTCCTTTG-3’
PPIA Reverse	5’-ACCACCCTGACACATAAACCCTG-3’
**CXCR7 promoter amplification primers**	
Forward (+ KpnI linker)	5’- CGTTGGTACCATGGGGTTTCACCATGTTGGC-3’
Reverse (+ XhoI linker)	5’- CGTTCTCGAGCTGGCTGGGCTGGCTG-3’
**ChIP AR-pulldown target RT-q-PCR primers**	
PSA Forward	5’-GCCTGGATCTGAGAGAGATATCATC-3’
PSA Reverse	5’-ACACCTTTTTTTTTCTGGATTGTTG-3’
ARE 1 Forward	5'-TACCCTCTCGTTTTCCCCCA-3'
ARE 1 Reverse	5'-CCTGAACTTGGCTGTCTCCT-3'
ARE 2 Forward	5'-CCCATGTGGCCTTTCTTCCA-3'
ARE 2 Reverse	5'GGGCCTGTCCTTCAGAGAAC-3'
ARE 3 Forward	5'-CTACAGCCACAGAAAGCGGA-3'
ARE 3 Reverse	5'-CTTTCTTGTGGGGGACCCTG-3'
ARE 4/5 Forward	5'-ATCCCCGTACATTTCCAGCG-3'
ARE 4/5 Reverse	5'-ACCTCGACTCTCCACAGCAT-3'
ARE 6 Forward	5'-CCGCTGGCAGTTTTCCTTTC-3'
ARE 6 Reverse	5'-ACTCTTCGTGTCACATCGGC-3'
**CRISPR gRNA sequences**	
CXCR7-gRNA 1	5’-TTTGCACGTGAGCTCGCCCA-3’
CXCR7-gRNA 2	5’-CATCTCGGCGTCCAGTGACC-3’
CXCR7-gRNA 3	5’-ACTGGACGCCGAGATGGCTC-3’

Supplementary Table S1: Sequences of primer sets utilized in this study and gRNA target sequences for CRISPR-Cas9 experiments.

Inhibition of androgen receptor promotes CXC-chemokine receptor 7-mediated prostate cancer cell survival

James J. Hoy, Georgios Kallifatidis, Diandra K. Smith, and Bal L. Lokeshwar

Supplementary Table S2: Antibody information

**Western blot**					
**Target**	**Host species**	**Supplier**	**Catalog number**	**Dilution**	**Blocker**
AR	Rabbit	Cell Signaling; Danvers, MA	5153	1:10,000	5% NFDM
Lamin A+C	Rabbit	Cambridge; United Kingdom	ab108595	1:5,000	5% NFDM
α-Tubulin	Rabbit	Abcam	ab4047	1:1,000	5% NFDM
CXCR7	Rabbit	Proteintech; Rosemont, IL	20423-1-AP	1:2,000	5% NFDM
phospho-Y1110 EGFR	Rabbit	Abcam	ab47370	1:5,000	5% BSA
EGFR (total)	Rabbit	Cell Signaling	2646S	1:5,000	5% BSA
phospho-ERK1/2	Mouse	Cell Signaling	9106S	1:5,000	5% NFDM
ERK1/2 (total)	Mouse	Cell Signaling	9107	1:5,000	5% NFDM
HRP-conjugated β-Actin	Rabbit	Cell Signaling	5125	1:10,000	5% NFDM
HRP-conjugated Rabbit	Goat	Cell Signaling	7074S	1:10,000	5% NFDM
HRP-conjugated Mouse	Horse	Cell Signaling	7076S	1:10,000	5% NFDM
**Chromatin Immunoprecipitation (ChIP) Assay**					
**Target**	**Host species**	**Supplier**	**Catalog number**	**Dilution**	**Blocker**
AR	Rabbit	EMD Millipore; Darmstadt, Germany	17-10489	2 μg / sample	N/A
**Proximity Ligation Assay (PLA)**					
**Target**	**Host species**	**Supplier**	**Catalog number**	**Dilution**	**Blocker**
CXCR7	Rabbit	GeneTex; Irvine, CA	GTX100027	1:300	PLA Kit supplied
ARRB1	Mouse	BioLegend; San Diego, CA	648602	1:15	PLA Kit supplied
ARRB2	Mouse	Santa Cruz Biotechnologies; Dallas, TX	sc-13140	1:60	PLA Kit supplied
Non-specific IgG	Mouse	Cell Signaling	5415	1:100	PLA Kit supplied

Supplementary Table S2: Details for antibodies used throughout this study. NFDM = non-fat dry milk, BSA = bovine serum albumin

Inhibition of androgen receptor promotes CXC-chemokine receptor 7-mediated prostate cancer cell survival

James J. Hoy, Georgios Kallifatidis, Diandra K. Smith, and Bal L. Lokeshwar

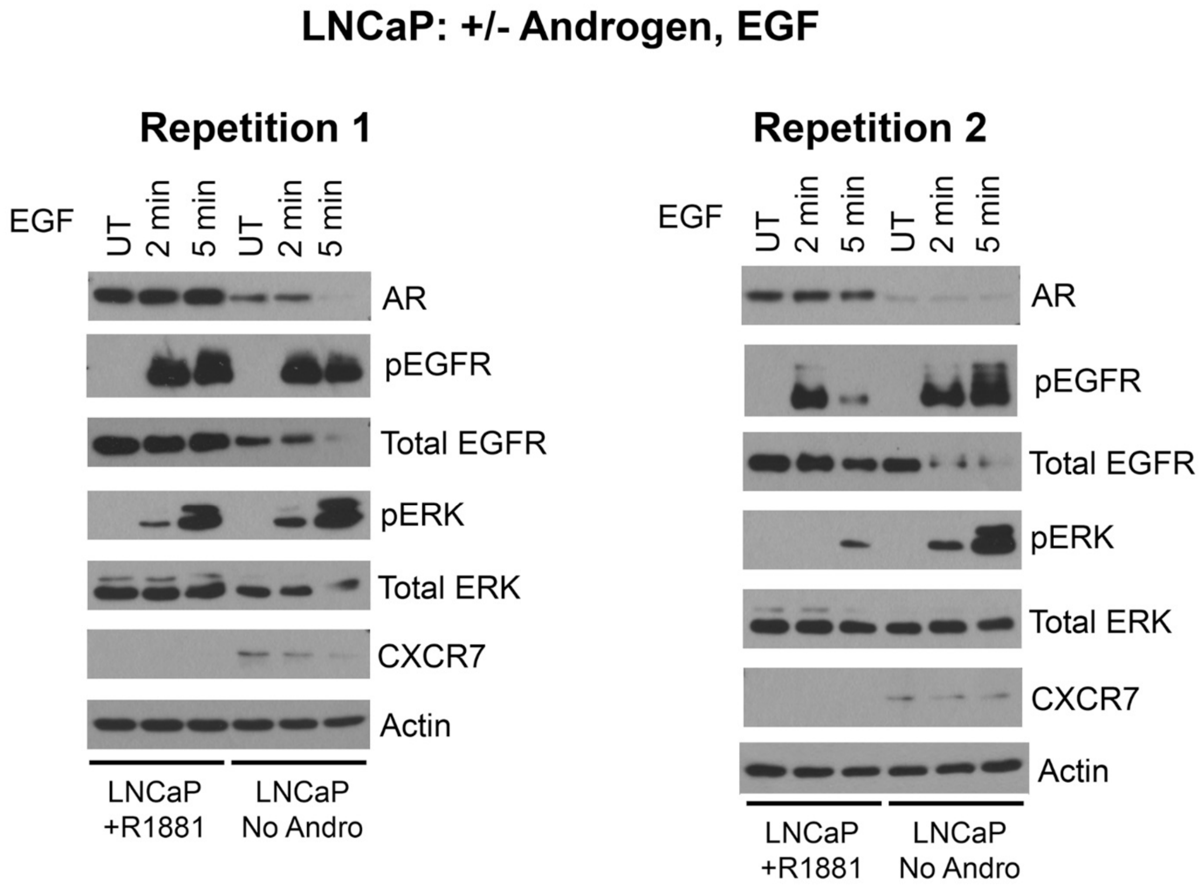

Supplementary Figure S1: Repetition of western blot experiment shown in figure 2a.

Inhibition of androgen receptor promotes CXC-chemokine receptor 7-mediated prostate cancer cell survival

James J. Hoy, Georgios Kallifatidis, Diandra K. Smith, and Bal L. Lokeshwar

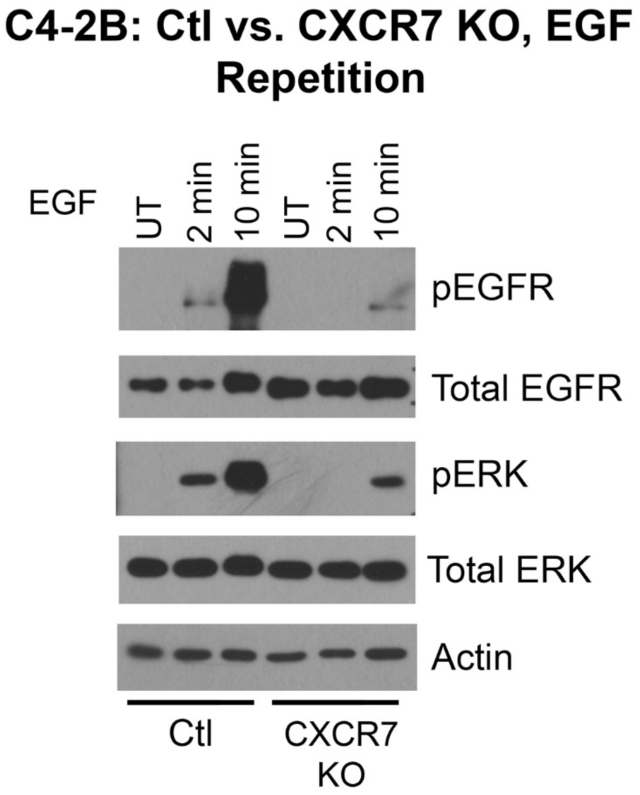

Supplementary Figure S2: Repetition of western blot experiment shown in figure 4b.

Inhibition of androgen receptor promotes CXC-chemokine receptor 7-mediated prostate cancer cell survival
James J. Hoy, Georgios Kallifatidis, Diandra K. Smith, and Bal L. Lokeshwar

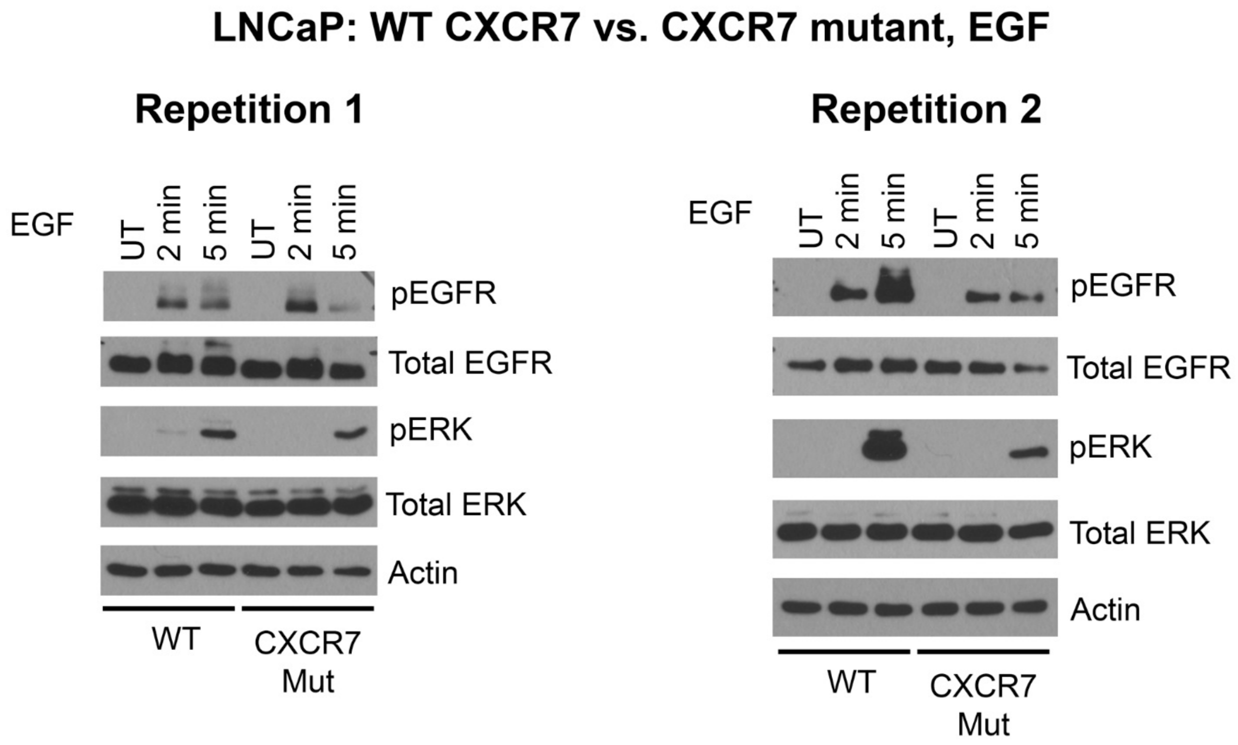

Supplementary Figure S3: Repetition of western blot experiment shown in figure 6b.

Inhibition of androgen receptor promotes CXC-chemokine receptor 7-mediated prostate cancer cell survival

James J. Hoy, Georgios Kallifatidis, Diandra K. Smith, and Bal L. Lokeshwar

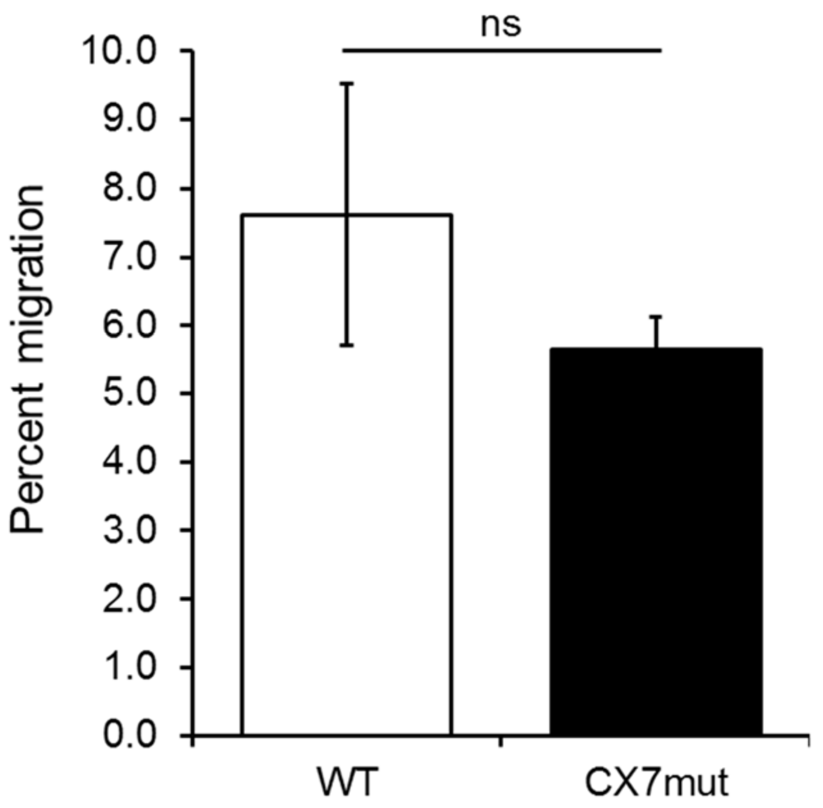

Supplementary Figure S4: CXCR7 mutation does not affect SDF-1α-mediated cell migration. Transwell migration assay of LNCaP WT and CXCR7-mutant cells towards an SDF-1α gradient. $n = 2$, mean ± SEM; ns = no significance; unpaired, two-tailed Student's t-test.
